# Increasing severity of anemia is associated with poorer 30-day outcomes for total shoulder arthroplasty

**DOI:** 10.1016/j.jseint.2021.02.001

**Published:** 2021-03-31

**Authors:** Matthew K. Doan, Jordan R. Pollock, M. Lane Moore, Jeffrey D. Hassebrock, Justin L. Makovicka, John M. Tokish, Karan A. Patel

**Affiliations:** a Mayo Clinic Alix School of Medicine, Mayo Clinic, Scottsdale, AZ, USA; b Department of Orthopedic Surgery, Mayo Clinic, Phoenix, AZ, USA

**Keywords:** Anemia, Shoulder, Arthroplasty, Degenerative disease, Osteoarthritis, Outcomes

## Abstract

**Background:**

Total shoulder arthroplasty (TSA) has increased in utilization over the past several decades. Anemia is a common preoperative condition among patients undergoing TSA and has been associated with poorer outcomes in other surgical procedures. To the best of our knowledge, no study has analyzed the association between anemia severity and TSA outcomes. Therefore, the purpose of this study is to determine the effects that increasing severity of anemia may have on the postoperative outcomes in patients receiving primary TSA.

**Methods:**

A retrospective analysis was performed using the American College of Surgeons National Surgery Quality Improvement Project database from the years 2015 to 2018. Current Procedure Terminology code 23472 was used to identify all primary TSA procedures recorded during this time frame. Patients with greater than 38% preoperative hematocrit (HCT) were classified as having normal HCT levels. Patients with HCT values between 33% and 38% were classified as having mild anemia. All patients with less than 33% HCT were classified as having moderate/severe anemia. Patient demographic information, preoperative risk factors, and postoperative outcomes were compared among the 3 cohorts. A multivariate logistic regression including demographic factors and comorbidities was performed to determine whether increasing severity of anemia is independently associated with poorer postoperative outcomes.

**Results:**

Of the 15,185 patients included in this study, 11,404 had normal HCT levels, 2962 patients were mildly anemic, and 819 patients had moderate to severe anemia. With increasing severity of anemia, there was an increased average hospital length of stay (1.6 vs. 2.1 vs. 3.0 days, *P* < .001), rate of readmissions (2.3% vs. 4.8% vs. 7.0%, *P* < .001), and rate of all reoperations (1.1% vs. 1.8% vs. 3.1%, *P* < .001). There was a statistically significant increase in both minor (1.9% vs. 2.7% vs. 4.4%, *P* < .001) and major (1.2% vs. 2.4% vs. 4.3%, *P* < .001) postoperative complication rates as well. Multivariate analysis identified anemia as an independent predictor of readmissions, reoperations, minor complications, and major complications.

**Conclusion:**

We found increasing severity of anemia to be associated with progressively worse 30-day postoperative outcomes. This is consistent with the outcomes found for increasing severity of anemia in patients receiving other total joint procedures. Using preoperative HCT levels may be a useful tool for predicting the risk of postoperative complications in patients undergoing TSA. This information could be used to further optimize patient selection for primary TSA.

Total shoulder arthroplasty (TSA) has become a successful treatment for many conditions and has increased in utilization over the past several decades.^21^^,^^22^ In fact, TSA utilization is projected to grow by up to 800% from 2011 to 2030.^21^ This growth rate is greater than both hip and knee arthroplasty procedures.^21^ Given this tremendous projected growth over the next decade, it is important to thoroughly understand the risk factors associated with undergoing and performing the procedure.

Despite significant advancements in TSA,^23^ complications can occur, with some patients reporting minimal or no improvement, and some even experience worsening of symptoms.^4^^,^^7^ In addition, complications increase healthcare costs for patients, physicians, and hospitals.^8^^,^^12^ As such, improving patient outcomes and containing costs remain a focus among orthopedic surgeons.^16^^,^^28^ Identifying conditions that predispose to complications resulting from TSA can help guide clinical practice and are of substantial benefit for both patients considering surgery and surgeons performing these procedures. A comprehensive understanding of predisposing conditions can also help give patients a more realistic expectation of outcomes before deciding on surgery. This will help patients and physicians make more informed decisions when deciding on the risks and benefits of undergoing or performing a procedure.

One such condition that has been known to increase postsurgical complications is anemia.^18^ Anemia is a relatively common condition among patients undergoing elective orthopedic surgery with studies reporting a prevalence between 21% and 35%.^2^^,^^9^ Anemia has also been found to be a significant risk factor for postoperative complications in total knee and hip arthroplasty,^5^^,^^10^ but the impact of severity of anemia on outcomes of patients undergoing TSA has not been defined. Given the profound growth in the volume of TSA procedures performed in the United States, we sought to investigate the potential link between anemia severity and patient outcomes after surgery. Therefore, the purpose of this study was to analyze the effects that increasing severity of anemia may have on the postoperative outcomes of patients undergoing primary TSA.

## Methods

### Database and patient selection

A retrospective analysis of the American College of Surgeons National Surgical Quality Improvement Program (ACS NSQIP) database was performed in the present study. The ACS NSQIP is a national, multicenter program aimed at measuring and promoting improved quality of surgical care.^30^ The definition and methods for the collection of variables in this database have previously been explained in similar studies.^3^^,^^5^^,^^15^^,^^17^ All primary TSA procedures recorded between 2015 and 2018 were identified using Current Procedure Terminology code 23472. Based on similar categorical values used in other studies,^10^^,^^14^ patients were stratified into 1 of 3 cohorts depending on their preoperative hematocrit (HCT) levels. Patients with greater than 38% preoperative HCT were classified as having normal HCT levels. Patients with HCT values between 33% and 38% were classified as having mild anemia. All patients with less than 33% HCT were classified as having moderate to severe anemia. Owing to the deidentified nature of the ACS NSQIP data, institutional review board approval was not required for this study.

### Variables collected

Preoperative demographic factors and comorbidities available in the database were collected in this study. Specific demographic variables include age, sex, and body mass index, whereas specific preoperative comorbidities include previous or current history of congestive heart failure, chronic obstructive pulmonary disease, bleeding disorders, diabetes mellitus, hypertension requiring medication, chronic steroid usage, history of smoking, American Society of Anesthesiologists (ASA) Classification, and sepsis. Thirty-day readmission rates, 30-day reoperation rates (return to operating room [OR]), operative time, hospital length of stay, and the occurrence of major or minor complications were all used as postoperative outcome measures. Based on similar criteria set by previous studies,^5^^,^^15^ major complications were defined as acute life-threatening complications and include sepsis, septic shock, acute renal failure, pulmonary embolism, ventilator usage greater than 48 hours, unplanned intubation, myocardial infarction, cardiac arrest requiring cardiopulmonary resuscitation, and stroke with neurologic deficits. Minor complications were defined as non-acute life-threatening complications including urinary tract infections, pneumonia, presence of surgical site infection, wound dehiscence, and deep vein thrombosis requiring therapy.

### Statistical analysis

JMP (SAS Institute, Cary, NC, USA) and Microsoft Excel (Microsoft Corp, Redmond, WA, USA) were used to perform the statistical analysis required for this study. Analysis of variance was performed for continuous variables, whereas chi-square tests were used for categorical variables. Continuous data were expressed as means with standard deviations. A multivariate logistic regression analysis was subsequently performed to determine any associations between patient comorbidities, increasing severity of anemia, and postoperative outcomes. Only preoperative measures found to have statistical significance based on initial univariate analysis were included in the logistic regression. A *P* value < .05 was set as the criteria for statistical significance.

## Results

### Patient demographics

Of the 15,185 patients included in this study, 11,404 had normal HCT levels, 2962 patients were considered mildly anemic, and 819 patients were considered to have moderate to severe anemia. Preoperative patient characteristics, comorbidities, and functional statuses based on respective HCT levels are displayed in Table I. With increasing severity of anemia, patients were more likely to be woman, older, and functionally dependent. Patients with severe anemia had lower body mass index on average. Furthermore, increased severity of anemia was associated with higher rates of preoperative COPD, congestive heart failure, cancer, diabetes, dialysis, dyspnea, hypertension, renal failure, sepsis, smoking, steroid use, weight loss, and increased ASA classification [Table I].

**Table I:** Preoperative demographics and comorbidities by hematocrit level.

	No anemia	Mild	Moderate/Severe	*P* value
Sex (female)	5615 (49.2%)	l279 (76.9%)	634 (77.4%)	<.0001∗
Age (yr ± SD)	68.6 ± 9.4	71.4 ± 9.1	72.5 ± 10.3	<.0001∗
BMI (yr ± SD)	31.4 ± 6.7	31.1 ± 7.3	29.8 ± 7.3	<.0001∗
Functional status (dependent)	180 (1.6%)	115 (3.9%)	52 (6.4%)	<.0001∗
HX COPD	727 (6.4%)	254 (8.6%)	89 (10.9%)	<.0001∗
HX CHF	50 (0.44%)	31 (1.1%)	17 (2.1%)	<.0001∗
Cancer	20 (0.2%)	12 (0.4%)	11 (1.3%)	<.0001∗
Diabetes	1849 (16.2%)	690 (23.3%)	224 (27.4%)	<.0001∗
Dialysis	20 (0.2%)	23 (0.8%)	16 (2.0%)	<.0001∗
Dyspnea	706 (6.2%)	239 (8.1%)	97 (11.8%)	<.0001∗
Hypertension	7430 (65.2%)	l214 (74.8%)	629 (76.8%)	<.0001∗
Renal failure	1 (< 0.1%)	2 (0.1%)	5 (0.6%)	<.0001∗
Sepsis	38 (0.3%)	26 (0.9%)	19 (2.3%)	<.0001∗
Smoker	1320 (11.6%)	251 (8.5%)	95 (11.6%)	<.0001∗
Steroid use	497 (4.4%)	212 (7.2%)	59 (7.2%)	<.0001∗
Weight loss	16 (0.1%)	12 (0.4%)	3 (0.4%)	.02∗
ASA class (>2)	6184 (54.2%)	1984 (67.0%)	652 (79.6%)	<.0001∗

*ASA*, American Society of Anesthesiologists; *BMI*, body mass index.

∗ Statistically significant.

### Postoperative outcomes: hospital length of stay, readmissions, and reoperations

Based on univariate analyses, increasing severity of anemia was associated with longer hospital length of stays (1.5 vs. 2.1 vs. 3.0 days, *P* < .0001), higher rates of 30-day readmissions (2.3% vs. 4.8% vs. 7.0%, *P* < .0001), and higher rates of 30-day return to operating room (1.1% vs. 1.8% vs. 3.1%, *P* < .0001). These associations are further illustrated in Table II.

**Table II:** Length of stay, readmissions, and reoperations by hematocrit level.

	No anemia	Mild anemia	Moderate/Severe anemia	*P* value
Length of stay (d)	1.5 ± 3.5	2. 1 ± 2.4	3.0 ± 8.6	<.0001∗
30-d readmission	261 (2.3%)	141 (4.8%)	57 (7.0%)	<.0001∗
30-d return to OR	29 (1.1%)	54 (1.8%)	25 (3.1%)	<.0001∗

∗ Statistically significant.

### Postoperative outcomes: major, minor, and total complication rates

In terms of major complications, increasing severity of anemia was associated with higher rates of postoperative myocardial infarction (0.2% vs. 0.5% vs. 0.9%, *P* = .006), cardiac arrest (<0.1% vs. 0.1% vs. 0.4%, *P* = .04), unplanned reintubation (0.2% vs. 0.2% vs. 1.3%, *P* < .0001), and prolonged use of ventilator (0.1% vs. 0.2% vs. 0.6%, *P* = .02). With regards to minor complications, increasing severity of anemia was associated with increased rates of pneumonia (0.5% vs. 0.5% vs. 1.7%, *P* = .0008) and urinary tract infection (0.6% vs. 0.9% vs. 1.5%, *P* = .006) [Table III].

**Table III:** Postoperative outcomes by hematocrit level.

	No anemia	Mild anemia	Moderate/Severe anemia	*P* value
Major Complications				
Acute MI	26 (0.2%)	14 (0.5%)	7 (0.9"/o)	.006∗
Cardiac arrest	5 (< 0.1%)	2 (0.1%)	3 (0.4%)	.04∗
Sepsis	15 (0.1%)	8 (0.3%)	3 (0.4%)	.14
Septic shock	3 (<0. 1%)	2 (0.1%)	2 (0.2%)	.09
Stroke	9 (0.1%)	4 (0.1%)	0 (0%)	.34
Pulmonary embolism	27 (0.2%)	14 (0.5%)	3 (0.4%)	.12
Renal complication	14 (0.1%)	6 (0.2%)	3 (0.4%)	.23
Reintubation	19 (0.2%)	7 (0.2%)	11 (1.3%)	<.00001∗
Ventilator >48 h	12 (0.1%)	5 (0.2%)	5 (0.6%)	.02∗
Minor complications				
DVT	45 (0.4%)	7 (0.2%)	7 (0.9%)	.06
Pneumonia	54 (0.5%)	16 (0.5%)	14 (1.7%)	.0008∗
Urinary tract infection	64 (0.6%)	27 (0.9%)	12 (1.5%)	.006∗
Wound disruption	4 (<0. 1%)	3 (0.1%)	1 (0.1%)	.31
Surgical site infection	48 (0.4%)	16 (0.5%)	3 (0.4%)	.66

∗ Statistically significant.

### Multivariate logistic regression analysis

HCT levels, sex, diabetes, smoking, functional status, congestive heart failure, chronic obstructive pulmonary disease, dialysis, dyspnea, hypertension, sepsis, steroid use, and ASA classification were all found to have statistical significance based on univariate analysis and were thus included in the multivariate analysis. Multivariate analysis found increasing severity of anemia to be independently associated with increased rates of minor complications, major complications, readmissions, and reoperations [Table IV]. More specifically, patients with mild anemia were not associated with increased rates of minor complications (OR 1.09, 95% confidence interval [CI], 0.80-1.47, *P* = .59), but patients with moderate/severe anemia were found to be correlated with increased minor complications (OR 1.79, 95% CI, 1.20-2.68, *P* = .005). Both patients with mild anemia (OR 1.49, 95% CI, 1.03-2.16, *P* = .03) and moderate/severe anemia (OR 2.43, 95% CI, 1.51-3.93, *P* = .0003) were independently associated with increased rates of major complications. Patients with mild anemia (OR 1.92, 95% CI, 1.53-2.39, *P* < .0001) and moderate/severe anemia (OR 2.45, 95% CI, 1.79-3.36, *P* < .0001) also had increased risk of readmissions. Finally, both mild (OR 1.78, 95% CI, 1.26-2.51, *P* = .0009) and moderate/severe anemia (OR 2.62, 95% CI, 1.63-4.20, *P* < .0001) were associated with increased risk of reoperation.

**Table IV:** Multivariate analysis of comorbidities in association with outcomes.

	Minor complications (OR with ?)	Major complications (OR with ?)	Readmissions (OR with ?)	Reoperations (OR with ?)
No anemia	Ref	Ref	Ref	Ref
Mild anemia	1.09 (0.80.1.47);	1.49 (1.03-2.16);	1.92 (1.53-2.39);	1.78 (1.26-2.51);
	*P* = .59	*P* = .034	*P* < .0001	*P* = .0009
Moderate/Severe anemia	1.79 (1.20.2.68);	2.43 (1.51-3.93);	2.45 (1.79-3.36);	2.62 (1.63-4.20);
	*P* = .005	*P* = .0003	*P* < .0001	*P* < .0001
Sex (female)	0.85 (0.67-1.22);	0.70 (0.51-0.97);	0.79 (0.65-0.96);	0.48 (0.36-0.65);
	*P* = .20	*P* = .031	*P* = .02	*P* < .000 1
Diabetes	0.90 (0.67-1.22);	1.40 (0.99-1.99);	1.08 (0.86-1.37);	1.03 (0.73-1.45);
	*P* = .51	*P* = .058	*P* = .49	*P* = .87
Smoking	1.19 (0.83-1.72);	I.I0 (0.66-1.84);	1.4 1 (0.79-1.51);	I.SO (0.99-2.25);
	*P* = .34	*P* = .70	*P* = .02	*P* = .053
Functional status (partial/full	1.39 (0.77-2.48);	0.92 (0.40-2.12);	2.27 (1.54-3.35);	2.49 (1.40-4.44);
dependence)	*P* = .27	*P* = .84	*P* < .0001	*P* = .006
Congestive heart failure	2.38 (1.09-5.19);	1.56 (0.54-4.50);	1.98 (1.01-3.87);	1.94 (0.74-5.13);
	*P* = .029	*P* = .41	*P* = .046	*P* = .18
Chronic obstructive	1.5I (1.04-2.19);	I.SI (0.95-2.4 1);	1.48 (1.09-1.99);	1.03 (0.63-1.67);
Pulmonary disease	*P* = .029	*P* = .081	*P* = .011	*P* = .91
Dialysis	1.80 (0.54-5.97);	0.72 (0.09-5.54);	1.68 (0.65-4.33);	2.02 (0.59-6.85);
	*P* = .34	*P* = .75	*P* = .29	*P* = .26
Dyspnea	1.29 (0.87-1.89);	1.26 (0.78-2.03);	1.09 (0.79.1.5I);	1.85 (1.20.2.85);
	*P* = .20	*PP* = .35	*P* = .60	*P* = .0056
Hypertension	0.92 (0.70.1.2 1);	0.85 (0.59-1.23);	1.09 (0.87-1.38);	1.44 (1.01-2.06);
	*P* = .54	*P* = .40	*P* = .45	*P* = .042
Sepsis	3.45 (1.53-7.78);	2.89 (1.01-8.28);	1.63 (0.69-3.84);	2.15 (0.66-7.03);
	*P* = .003	*P* = .049	*P* = .27	*P* = .20
Steroid use	1.06 (0.66-1.70);	1.39 (0.80.2.42);	1.38 (0.98-1.96);	1.02 (0.57-1.84);
	*P* = .82	*P* = .18	*P* = .07	*P* = .95
ASA class (>2)	2.09 (1.55-2.82);	3.47 (2.17-5.54);	1.88 (1.48-2.40);	1.41 (1.00.1.99);
	*P* < .0001	*P* < .0001	*P* < .0001	*P* = .052

*ASA*, American Society of Anesthesiologists.

Ref indicates reference values for other patient cohorts.

## Discussion

As shoulder arthroplasty becomes increasingly common in the United States,^22^ a comprehensive understanding of predisposing risk factors for complications becomes essential. Common complications of TSA include periprosthetic fracture, glenoid and humeral component loosening, infections, and rotator cuff tears.^4^ In addition, common risk factors include age, ASA classification, diabetes, chronic obstructive pulmonary disease, congestive heart failure, dialysis, history of a bleeding disorder, and operative time.^11^ However, little is known about the role that increasingly severe anemia may play in predisposing a patient to postoperative complications. Our study demonstrated that increasing severity of anemia is independently associated with increased rates of minor complications, major complications, hospital readmissions, and reoperations through multivariate analysis. These findings are consistent with other studies examining anemia in knee and hip arthroplasty.^10^^,^^19^

We found increasing severity of anemia to be associated with increased rates of minor complications, major complications, 30-day readmissions, 30-day reoperations, and increased hospital length of stay which are factors that should be considered when performing a TSA on a patient with anemia. These poor patient outcomes included significantly higher rates of postoperative myocardial infarction, cardiac arrest, unplanned reintubation, prolonged use of ventilators, increased rates of pneumonia, and increased rates of urinary tract infection. While the benefits of TSA may outweigh the potential complications, appropriate patient counseling on risks and benefits of TSA for patients with preoperative anemia should be emphasized.

Preoperative anemia was found to significantly increase the risk of postoperative acute myocardial infarction and cardiac arrest, with severely anemic patients having more than a 400% greater risk of myocardial infarction or cardiac arrest than patients with no anemia. In addition, patients with anemia are more likely to have worse outcomes and higher mortality when these complications occur.^6^^,^^26^ The increase in risk of postoperative acute myocardial infarction and cardiac arrest has been noted in other studies. A study by Oldeji et al^20^ found a similar relationship between presurgery anemia and the risk of myocardial infarction after TSA with an OR of 1.62. Interestingly, mild anemia was not associated with an increased risk for minor complications, but it was associated with a greater risk for major complications. This may suggest that any degree of anemia in a preoperative patient with TSA is a very important risk factor and should be closely monitored owing to the increased risk of the devastating consequences of a major complication.

Reducing complications during TSA helps improve patient outcomes and satisfaction, but it also contains costs for hospitals.^27^ Recent healthcare policy has been created and implemented in an attempt to improve patient satisfaction and contain costs, such as the Medicare Access and Chip Reauthorization Act of 2015 and the creation of subsequent value-based payment models such as the Merit-based Incentive Payment System which incentivize quality over quantity.^24^^,^^29^ The findings in our study could prove useful as these new incentive programs are incorporated into orthopedic practice. For example, severity of anemia could help more accurately perform risk prediction in models such as bundled payments;^25^ which require accurate risk-prediction models to remain cost-effective. For example, hospitals with higher 30-day readmission rates are significantly more likely to incur reimbursement penalties.^13^ As per our study results, we believe factors such as severity of anemia in patients with TSA should be included in preoperative risk prediction as to not unfairly penalize surgeons who are providing otherwise quality, value-based care.

One significant complication of any surgery is hospital readmission. The 30-day readmission rate in orthopedics due to complications continues to be an area of potential cost-containment, as current 30-day readmission rates are estimated to be between 4.8% and 6.0%.^1^ Based on univariate analyses in our study, increasing severity of anemia was associated with significantly longer hospital length of stays, higher rates of 30-day readmissions, and higher rates of 30-day return to the operating room, indicating anemia could prove to be a costly presurgical condition. The financial risk associated with operating on patients with preoperative anemia is apparent and should be considered.

Our study has several notable limitations. First, we defined severity of anemia by HCT, which is not an absolute indicator for anemia. However, the HCT classifications we used are based off previously verified studies examining anemia in preoperative patients. An inherent limitation of using the NSQIP database is the comorbidities and other variables of interest were limited to the variables included in the database, and the accuracy of the data is dependent on the validity of the information entered into the electronic medical record. In addition, patient selection is not randomized, and the database is not comprehensive. As a result, our multivariate analysis did not include all possible comorbidities and demographic factors. We also could not account for unreported differences in preoperative conditions or all surgical experiences and complications given the retrospective nature of this study. Finally, outcome measures in this study may not encompass the potential long-term complications as data were limited to 30-day postoperative results. Despite these limitations, the NSQIP database is widely used and provides a relatively large and nationally representative sample for examination of a clinically relevant topic in TSA.

## Conclusion

As the population continues to age, TSA will continue to increase in utilization dramatically. Anemia is a relatively common condition found in patients undergoing elective orthopedic procedures including TSA. In this study, increasing severity of anemia was found to be associated with progressively worse 30-day outcomes. Specifically, increasing severity of anemia is an independent predictor of increased 30-day readmission, reoperation, major complications, and minor complications based on multivariate analysis performed using the variables available. These findings suggest that using preoperative HCT levels may be a useful tool for predicting postoperative outcomes and optimizing patient selection for primary TSA. Further studies are warranted, however, to analyze the long-term associations between anemia and outcomes for primary TSA.

## Disclaimers:

*Funding:* No funding was disclosed by the author(s).

*Conflicts of interest:* John M. Tokish is a consultant for Arthrex and receives royalties and grant support; he is also a consultant for Depuy Mitek.

The other authors, their immediate families, and any research foundation with which they are affiliated did not receive any financial payments or other benefits from any commercial entity related to the subject of this article.
